# Preparation and Characterization of Small-Size and Strong Antioxidant Nanocarriers to Enhance the Stability and Bioactivity of Curcumin

**DOI:** 10.3390/foods13233958

**Published:** 2024-12-08

**Authors:** Shanshan Tie, Yujin Yang, Jiawei Ding, Yanyan Li, Mengmeng Xue, Jianrui Sun, Fang Li, Qiuxia Fan, Ying Wu, Shaobin Gu

**Affiliations:** College of Food and Bioengineering, Henan University of Science and Technology, Luoyang 471023, China; tieshanshan@haust.edu.cn (S.T.);

**Keywords:** curcumin, nanoparticles, procyanidins, Mannich reaction, stability

## Abstract

The purpose of this study was to design nanocarriers with small-size and antioxidant properties for the effective encapsulation of curcumin. Here, procyanidins, vanillin, and amino acids were used to successfully prepare nanocarriers of a controllable size in the range of 328~953 nm and to endow antioxidant ability based on a one-step self-assembly method. The reaction involved a Mannich reaction on the phenolic hydroxyl groups of procyanidins, aldehyde groups of vanillin, and amino groups of amino acids. Subsequently, curcumin nanoparticles were prepared by loading curcumin with this nanocarrier, and the encapsulation efficiency of curcumin was 85.97%. Compared with free curcumin, the antioxidant capacity and photothermal stability of the embedded curcumin were significantly improved, and it could be slowly released into simulated digestive fluid. Moreover, using the corticosterone-induced PC12 cell injury model, the cell viability increased by 23.77% after the intervention of curcumin nanoparticles, and the cellular antioxidant capacity was also significantly improved. The nanoparticles prepared in this work can effectively improve the solubility, stability, and bioactivity of curcumin, which provides a reference for the embedding and delivery of other hydrophobic bioactive compounds.

## 1. Introduction

Different bioactive compounds, found in natural resources such as vegetables, fruits, and nuts, can effectively promote human health and prevent various chronic diseases. Curcumin (Cur), as a highly lipophilic plant polyphenol, is extracted from the rhizome of *Curcuma longa* L. in the ginger family [1,2]. It is mainly composed of a benzene ring, a pyran ring, and ketone carbonyl chemical structures, which make it possess many beneficial biological activities, such as antioxidant, neuroprotective, chemopreventive, and immunomodulatory effects [3]. Due to these health effects, Cur may be a candidate for potential use in chronic neurological diseases, such as depression and neuroinflammation [4,5]. However, the biological effects of Cur are severely constrained due to its poor aqueous solubility, physicochemical instability, poor oral bioavailability, and low targeting effect, as well as its 89% excretion rate in its original form after high-dose oral administration [6,7]. Therefore, the question of how to improve the stability of Cur and maximize its biological effect is an urgent problem to be solved.

In terms of overcoming the limitations faced by Cur as a hydrophobic active compound, the construction of nanocarriers for loading and delivering Cur has become an effective way to improve the stability and bioavailability of Cur. Different nanostructures, such as nanoparticles, liposomes, nanoemulsions, nanomicelles, and nanogels, prepared by self-assembly, anti-solvent precipitation, emulsification, low-temperature solidification, and other methods are widely used as nanocarriers [8,9,10]. For example, Liu et al. used a self-assembly and electrostatic deposition method to prepare zein-sodium caseinate nanoparticles for loading Cur, which effectively improved the chemical degradation resistance, radical scavenging capacity, and bio-accessibility of Cur [11]. These nanocarriers all possess the advantages of easy construction, reproducibility, non-toxicity, biocompatibility, and biodegradation [12]. Moreover, an ideal nanocarrier should also have a synergistic effect with bioactive compounds and a suitable particle size, which can increase their targeting performance and residence time.

Procyanidins (PCs) are sustainable polyphenolic compounds with amphipathic properties, network-forming abilities, biocompatibility, and health effects. This makes their use possible as materials for the preparation of nanocarriers [13,14]. PCs with a strong antioxidant capacity are also stable in the stomach, and using them as wall materials can effectively protect bioactive compounds and exert a synergistic antioxidant effect. PCs are rich in aromatic rings and hydroxyl groups and can be combined with common macromolecules such as proteins and polysaccharides, and this is an important way to form nanocarriers. However, these macromolecules are heterogeneous, and unexpected risks may arise after their ingestion in the body, such as the immunogenicity of proteins, which will bring adverse effects when used to prepare PC nanocarriers [15]. Amino acid, as a small-molecule substance, is the basic component unit of protein. Amino acids have the advantages of simple and known chemical structures, abundant sources, safety and reliability, and no immunogenicity. This provides potential possibilities for the subsequent synthesis of PC-based nanoparticles. The Mannich reaction is a carbon−carbon bond formation reaction that relies on one pot of three-component reactants, i.e., amine, aldehyde, and ketone [16]. In this study, PCs, amino acids, vanillin-containing phenolic hydroxyl, and aldehyde structures dispersed in water underwent a Mannich reaction and generated linear PC derivatives. Intermolecular hydrogen bonding and π–π stacking forces increase the entanglement and interaction between different molecules, ultimately promoting the self-assembly of these oligomers into PC-based nanoparticles [17].

In this work, nanocarriers with controllable sizes and antioxidant capacities were synthesized by a Mannich reaction for the encapsulation and delivery of Cur, and the intervention effect of these prepared Cur nanoparticles on PC12 cell injury, induced by corticosterone (CORT), was evaluated. Firstly, the size of nanocarriers was accurately controlled by adjusting the proportion of PCs, amino acids, and vanillin. This optimized nanocarrier was used to load Cur, and the morphology, encapsulation efficiency, antioxidant capacity, photothermal stability, and simulated digestion of Cur nanoparticles were characterized. Subsequently, the biocompatibility and cellular antioxidant activity of Cur nanoparticles were analyzed by using the PC12 cell injury model. The results proved that the nanocarrier designed in this study can effectively improve the stability and biological effect of loaded Cur, which can give a reference for the embedding and delivery of other hydrophobic bioactive ingredients.

## 2. Materials and Methods

### 2.1. Materials

Curcumin (Cur), Lysine, Glutamic acid, Cystine, and vanillin were purchased from Shanghai Macklin Biochemical Technology Co., Ltd. (Shanghai, China). Procyanidins (PCs) were obtained from Tianjin Jianfeng Natural Product R&D Co., Ltd. (Tianjin, China). Corticosterone (CORT) was obtained from Shanghai D&B Biotechnology Co., Ltd. (Shanghai, China). Dulbecco’s modified Eagle medium (DMEM), fetal bovine serum, penicillin-streptomycin, and 3−(4,5−dimethylthiazol−2−yl)−2,5−diphenyltetrazolium bromide (MTT) were purchased from Shanghai Beyotime Biotechnology Co., Ltd. (Shanghai, China). Total superoxide dismutase (T-SOD), catalase (CAT), and malonic dialdehyde (MDA) detection kits were purchased from the Nanjing Jiancheng Bioengineering Institute (Nanjing, China).

### 2.2. Fabrication of Nanocarriers

The nanocarriers (NCs) were prepared by a Mannich reaction using PCs, vanillin, and amino acids as raw materials. Specifically, PCs and vanillin were evenly dispersed in 30 mL distilled water; then, lysine (cysteine or glutamic acid), accounting for 1/10 of the mass of PCs, was added; and the whole process was stirred for 24 h. Subsequently, the above solution was centrifuged at 10,000 rpm for 10 min to obtain NCs and resuspended in water for later use. The main parameters of NCs−1~NCs−8 prepared by different concentrations of PCs, vanillin, and amino acids are shown in Table 1.

### 2.3. Particle Size and Structure Characterization of Nanoparticles

The particle size, polydispersity index (PDI), and size distribution of NCs−1~NCs−8 nanoparticles were recorded by a Bettersize 2600 instrument (Better, Shanghai, China).

The possible molecular interactions among PCs, vanillin, and lysine in the optimized NCs−1 nanoparticles were analyzed by a VERTEX70 Fourier-transform infrared spectrometer (FTIR, Bruker, Germany) at the spectral range of 4000–400 cm^−1^. The ultraviolet-visible (UV-Vis) absorption spectra of PCs and NCs−1 were recorded by a UV2600 absorption spectrophotometer (Shimadzu, Japan). The crystalline structures of PCs and NCs−1 were obtained by an X-ray diffraction instrument (XRD, Rigaku SmartLab SE, Japan), and samples were scanned at a speed of 10°/min in the range of 10 to 80°.

### 2.4. Preparation and Characterization of Cur Nanoparticles

Cur nanoparticles (NPs) were prepared by an anti-solvent rotary evaporation method, with Cur as the active ingredient and NCs−1 as carriers. Specifically, Cur ethanol solution (2 mg/mL) was dispersed in an equal volume of NCs−1 carrier solution (2 mg/mL), stirred at the dark for 24 h, and then ethanol was eliminated by rotary evaporation at 45 °C to obtain Cur NPs.

The absorbance of Cur solutions with different concentrations (1, 3, 5, 8, 10, and 13 μg/mL) at 426 nm was measured, and the standard curve was obtained. The solution of Cur NPs was centrifuged at 8000 rpm for 10 min. The absorbance of the supernatant at the same wavelength was determined, and the encapsulation efficiency of Cur was calculated based on the standard curve [18,19]. The surface morphology of Cur NPs was recorded by TM3030Plus SEM. The FTIR spectra, UV-Vis spectra, and crystal structures of Cur and Cur NPs were recorded by an FTIR instrument, a UV2600 spectrophotometer, and an XRD instrument, respectively.

### 2.5. Antioxidant Ability

The antioxidant ability of the positive control (Vc), Cur, NCs, and Cur NPs were analyzed by DPPH and ABTS radical scavenging experiments. The detailed steps referred to the method reported by Huang et al. [20].

### 2.6. Light and Thermal Stability Experiments

To evaluate whether the prepared nanoparticles could effectively protect Cur, the stability of the samples under UV radiation and high temperature conditions was monitored. Cur and Cur NPs solution in transparent centrifuge tubes were placed in a UV radiation chamber for 0, 20, 40, 60, and 90 min, and the level of Cur in the samples was analyzed. In the thermal stability experiment, Cur and Cur NPs were placed in a water bath at 25, 37, and 50 °C for 30 min and cooled to room temperature, and then the content of Cur was determined.

### 2.7. In Vitro Simulated Digestion Experiment

The digestive fluid is mainly composed of artificial gastric fluid and intestinal fluid. Cur NPs (1 mL) was mixed with simulated gastric fluid (85 mM sodium chloride and 3 g/L pepsin, 10 mL), and pH was regulated to 2.0. The mixed suspension was incubated at 37 °C for 2 h, and samples were obtained at regular intervals to measure the absorbance value of Cur. Subsequently, the above mixture (1 mL) was added to intestinal fluid (10 mL), regulated to a pH of 7.0 and cultured for 2 h. The content of Cur at different time points was determined by a microplate reader at 426 nm.

### 2.8. Cell Experiments

PC12 cells were placed in DMEM medium containing 10% fetal bovine serum and 1% double antibody in an incubator containing 5% CO_2_. The status of cells was observed daily, and cells in the logarithmic growth were used for the subsequent tests. Different contents of CORT (100, 200, 300, 400, 500, 600, 700, and 800 μM) were used to treat PC12 cells for 24 h, and the optimal concentration of cell viability damage was screened so as to obtain a CORT-induced PC12 cell injury model.

#### 2.8.1. Cytotoxicity Assay

The cell viability of the samples was recorded by an MTT assay. PC12 cells were inoculated in 96-well plates (100 μL) for 24 h, and fresh DMEM containing different contents of Cur, NCs, or Cur NPs (1, 2.5, 5, 10, 15, 20, and 40 μM) was replaced for continued incubation for 24 h. Subsequently, after treatment with 400 μM CORT for 24 h, 5 mg/mL of MTT (20 μL) was added to continue incubation for 4 h, and the supernatant was eliminated. Finally, dimethyl sulfoxide (150 μL) was added, and the OD_490_ value was analyzed to calculate the cell viability.

#### 2.8.2. Intracellular T-SOD, CAT and MDA Levels

PC12 cells seeded in 12-well plates were cultured with 10 μg/mL Cur, NCs, or Cur NPs, and then incubated with 400 μM CORT for an additional 24 h. The cell supernatants were collected, and the contents of T-SOD, CAT, and MDA in the DMEM medium were analyzed using the relevant assay kits.

### 2.9. Hemolysis Test

The hemocompatibility of NCs and Cur NPs was evaluated by using red blood cells from Kunming mice. Fresh blood was extracted from healthy mice, evenly mixed with an appropriate amount of anticoagulant, and centrifuged at 3000 rpm for 10 min. The red blood cells were repeatedly washed with normal saline until the supernatant was colorless. Subsequently, the supernatant was discarded, and 2% red blood cell suspension was prepared with normal saline for hemolysis test. The red blood cell suspension (1 mL) and 0.5 mL of samples were mixed and incubated at 37 °C for 2 h. The mixture was centrifuged, and the absorbance value of the supernatant at 545 nm was determined. Normal saline was used as a negative control, and an equivalent amount of 0.1% TritonX−100 was used as a positive control. The hemolysis rate (HR) of the sample was calculated according to the following formula:

HR (%) = (A_sample_ − A_negative_)/(A_positive_ − A_negative_) × 100%

where A_negative_, A_positive_, and A_sample_ represent the OD values of normal saline, TritonX−100, and the sample, respectively.

### 2.10. Statistical Analysis

The tests were performed at least three times. The obtained results were presented as mean ± SD and assessed by one-way ANOVA using SPSS 19.0 statistical software. *p* < 0.05 was the criterion of statistical significance.

## 3. Results and Discussion

### 3.1. The Particle Size Characterization of Size-Controlled Nanocarriers

**Figure 1:**
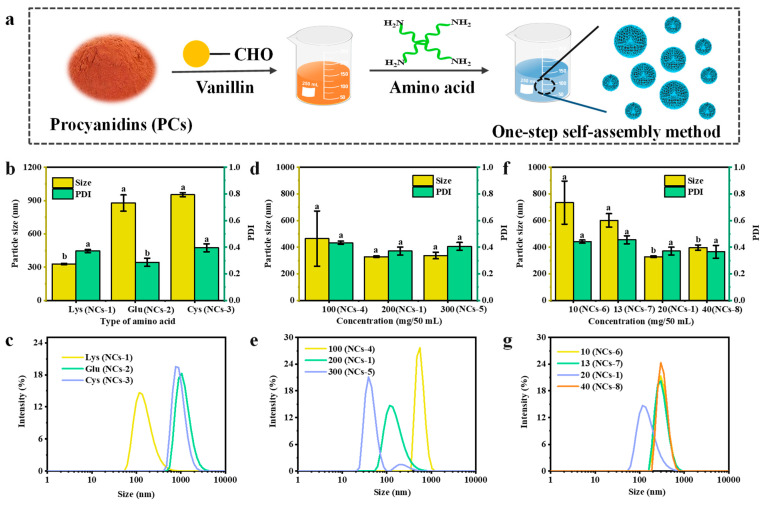
Preparation and particle size characterization of nanocarriers. (**a**) A schematic diagram of the preparation of NCs−1~NCs−8 using PCs, vanillin, and amino acids as raw materials. (**b**) Particle size, polydispersity index (PDI), and (**c**) particle size distribution of amino acid-dependent nanocarriers NCs−1~NCs−3. (**d**) Particle size, PDI, and (**e**) particle size distribution of PC-dependent nanocarriers NCs−1 and NCs−4~NCs−5. (**f**) Particle size, PDI, and (**g**) particle size distribution of vanillin-dependent nanocarriers NCs−1 and NCs−6~NCs−8. Note: the lower letters a, b, and c indicate that there are statistically significant differences between the samples.

As one of the important properties of nanocarriers, particle size not only determines the physico-chemical properties of nanocarriers, but also affects their biological properties, such as delivery, targeting, distribution, or residence time in organisms [21]. The molecular self-assembly method is actually a concentration- or species-dependent method that could be used to prepare size-controlled nanocarriers using PCs, vanillin, and amino acids as the raw materials (Figure 1a) [22].

The regulating effects of different concentrations of PCs and vanillin, or the types of amino acids, on the particle size of nanocarriers were studied, with the other two components remaining unchanged. As shown in Figure 1b, Figure 1c, and Table 1, in the amino acid group, NCs−1, NCs−2, and NCs−3 are prepared at 200 mg/50 mL PCs and 20 mg/50 mL vanillin with varying types of amino acids (Lys, Glu, or Cys, respectively). The corresponding sizes were 328.47 nm (PDI = 0.37) for NCs−1, 877.87 nm (PDI = 0.29) for NCs−2, and 952.99 nm (PDI = 0.40) for NCs−3, and all presented a normal distribution. In the PC-dependent group, the particle sizes of NCs−4, NCs−1, and NCs−5 were 463.96 nm (PDI = 0.43), 328.47 nm (PDI = 0.37), and 337.44 nm (PDI = 0.41), respectively, which were prepared from different concentrations of PCs (Figure 1d,e). The results showed that with an increase in PC content, the particle size of the nanocarriers first decreased and then increased. However, the particle size distribution range of NCs−5 was wide. Moreover, the particle size results in Figure 1f,g showed that, when the vanillin concentration was 10, 13, 20, and 40 mg/50 mL, the particle sizes of the prepared NCs−6, NCs−7, NCs−1, and NCs−8 were 734.63 nm (PDI = 0.44), 601.48 nm (PDI = 0.46), 328.47 nm (PDI = 0.37), and 397.02 nm (PDI = 0.37), respectively. A similar size distribution trend was observed for different contents of the vanillin-treated group. When the concentrations of PCs, vanillin, and Lys were 200, 20, and 50 mg/50 mL, the particle size of NCs−1 was smallest, at 328.47 nm, and thus, NCs−1 were selected for subsequent experiments.

### 3.2. Synthesis and Spectral Characterization of Nanocarriers

**Figure 2:**
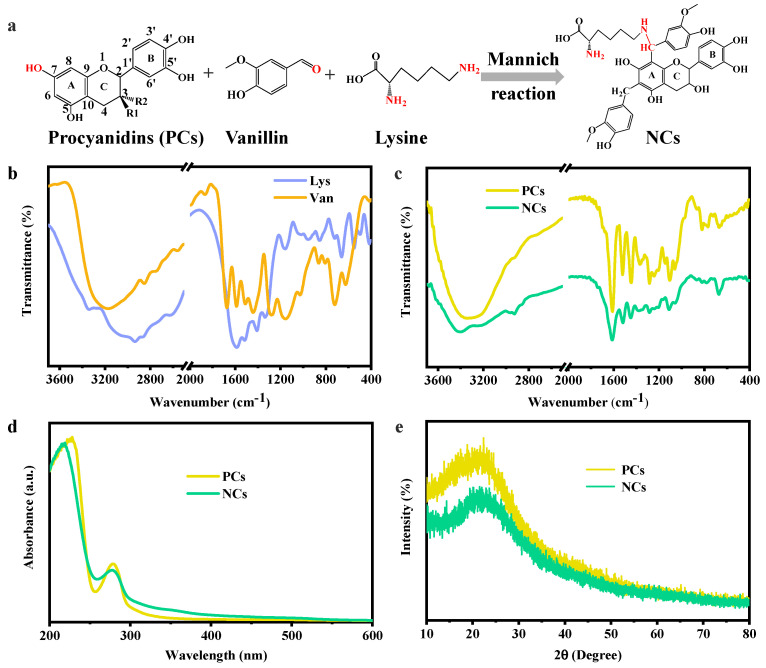
Formation and spectral characterization. (**a**) Schematic illustration of the reaction pathway of NCs. FTIR spectra of (**b**) vanillin (Van), Lys, (**c**) PCs, and NCs. (**d**) UV-Vis spectra and (**e**) crystal structure of PCs and NCs.

A nanocarrier was synthesized using a “one-step” self-assembly method based on the Mannich reaction among −OH of PCs, −CHO of vanillin, and NH_2_ of Lys (Figure 2a). The functional groups of PCs, vanillin, and Lys and the prepared NCs were analyzed by FTIR spectroscopy, and the results are shown in Figure 2b,c. The absorption peak of PCs at 3340 cm^−1^ belonged to the O−H stretching vibration of phenolic hydroxyl groups [23]. For Lys, the peak at 3341 cm^−1^ was attributed to the stretching vibration of N−H groups, and the absorption peaks at 2936 and 2872 cm^−1^ corresponded to the stretching vibration of CH_2_ and CH_3_ groups [24]. These peaks all appeared in the as-prepared NCs and shifted to 3399, 2928, and 2864 cm^−1^, respectively. However, the characteristic peaks of Lys at 1585 and 1516 cm^−1^ belonging to C=O stretching vibration and N−H bending vibration were almost overlapped with the peaks of PCs, and were, thus, hardly discernible in NCs. For NCs, the main peaks at 1614, 1518, and 1448 cm^−1^ were attributed to the stretching vibrations of the aromatic ring (C−C/C=C) of PCs [20]. Moreover, the deformation vibration of C−H bonds in the benzene ring at 1146, 820, and 766 cm^−1^ completely disappeared, which was accompanied by the enhancement of the peak at 671 cm^−1^, indicating the formation of methylene bridges.

The results of the UV-Vis spectra showed that PCs and NCs had two absorption peaks in the range of 200-600 nm (Figure 2d). The peaks of PCs at 226 and 279 nm corresponded to the electron transition from the far-ultraviolet region to the near-ultraviolet region (π → π*) on benzene ring and the near-ultraviolet region (n → π*) of A and B rings’ conjugated structure, respectively [25]. Compared with PCs, the two absorption peaks of NCs prepared by PCs showed a blue shift, which was related to the changes of surrounding microenvironment and the decrease in auxochrome (i.e., −OH and −NH_2_) level caused by the Mannich reaction among PCs, vanillin, and Lys. Moreover, as shown in Figure 2e, PCs have a diffraction peak at 2θ value of 22.22°, which indicate an amorphous crystal state. The prepared NCs showed a similar peak at the value of 21.80°, indicating that the intermolecular interaction did not change the crystal diffraction mode of NCs. Therefore, a nanocarrier with a small size was successfully synthesized according to the FTIR spectra, UV−vis spectra, and XRD results.

### 3.3. Preparation and Characterization of Cur-Loaded Nanoparticles

**Figure 3:**
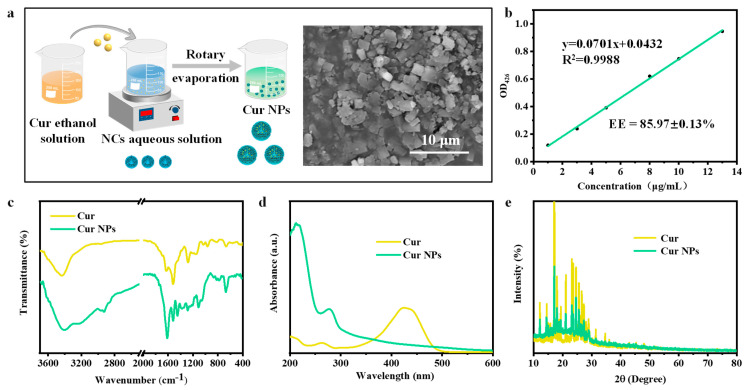
Preparation and characterization of Cur NPs. (**a**) A schematic diagram of preparation, SEM image, and (**b**) embedding efficiency (EE) of Cur NPs. (**c**) FTIR spectra, (**d**) UV−vis spectra and (**e**) crystal structure of Cur NPs.

The combination of anti-solvent effect and rotary evaporation is one of the effective methods for preparing hydrophobic bioactive compound-based nanoparticles. The alcoholic solution of hydrophobic compound−Cur was added dropwise to the aqueous solution of NCs under magnetic stirring by a microinjection pump, and Cur NPs were prepared by an anti-solvent rotary evaporation method (Figure 3a). The obtained Cur NPs are basically square with a uniform state.

Embedding efficiency was also a main index to assess whether the designed nanocarriers could be used to load Cur. According to the standard curve of Cur (y = 0.0701x + 0.0432, R^2^ = 0.9988), the embedding efficiency of Cur NPs was 85.97% (Figure 3b). FTIR spectra of Cur in Figure 3c show that the peaks at 3425, 1630, 1520, and 1279 cm^−1^ corresponded to the stretching of phenolic hydroxyl groups, mixed tensile vibrations of C=C and C=O, C−O and C−C vibrations, and aromatic C−O stretching vibrations, respectively [26,27]. These peaks of Cur disappeared after being loaded into NCs, and only the peaks at 3398, 1614, and 1518 cm^−1^ of NCs were observed in the spectra of Cur NPs, indicating that Cur loaded into nanocarriers would form hydrophobic cores, which led to the masking of their characteristic peaks. This observed phenomenon was similar to that described in the report by Lin et al., who reported that the peak of pure Cur disappeared when Cur was encapsulated in nanoparticles [8]. As shown in Figure 3d, Cur has two absorption peaks at 263 and 424 nm in the near-ultraviolet and visible regions. However, after Cur was encapsulated, the absorption peaks of the obtained Cur NPs were found at 212 and 277 nm, and the peaks of Cur were not obvious, which was consistent with the results obtained by FTIR. Moreover, due to the high crystal structure of pure Cur, multiple diffraction peaks were found at values of 12.18°, 14.54°, 17.08°, 18.08°, 19.38°, 21.14°, 23.22°, 24.62°, 25.60°, 26.60°, 27.36°, and 28.96° (Figure 3e) [28]. NCs were in an amorphous state, and Cur NPs obtained by loading Cur into these NCs also showed similar diffraction peaks at 2θ values of 12.18°, 14.54°, 17.14°, 18.08°, 19.36°, 21.12°, 23.22°, 24.60°, 25.60°, 26.58°, 27.36°, and 28.96°. But the peak intensities of these diffraction peaks were obviously reduced, and the positions were partially shifted. Jiang et al. found that β−cyclodextrin nanoparticles loaded with Cur also showed similar changes in their crystal structure [29]. These results all proved that Cur was successfully loaded into the prepared nanocarriers.

### 3.4. Antioxidant Capacity Analysis

**Figure 4:**
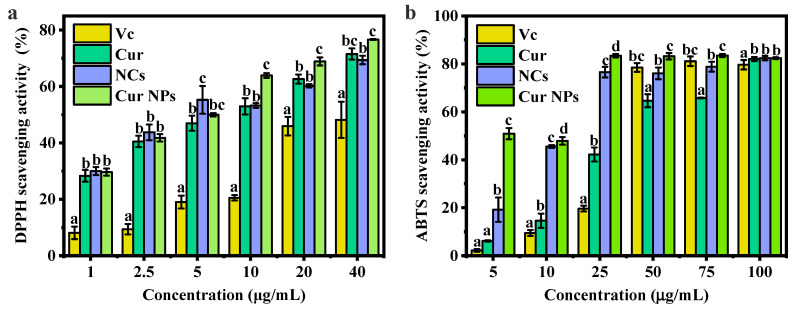
Antioxidant capacity experiment. (**a**) DPPH and (**b**) ABTS radical scavenging activities for Cur, NCs, and Cur NPs. Note: the lower letters a, b, and c indicate that there are statistically significant differences between the samples.

To evaluate the antioxidant capacities of Cur NPs, DPPH and ABTS radical scavenging experiments were conducted. DPPH, as a stable radical, has a maximum absorption value at 517 nm in ethanol solution, and the solution is purple color. The absorbance results of the positive control—Vc, Cur, NCs, and Cur NPs at 517 nm showed that the scavenging ability of DPPH radicals was positively correlated with their concentration, and the dose-dependent phenomenon appeared from 0 to 40 μg/mL (Figure 4a). Compared with Vc, Cur dispersed in ethanol solution at a low concentration (≤5 μg/mL) had high antioxidant ability. When the concentration of Cur or NCs was 40 μg/mL, the radical scavenging percentage of Cur NPs was 76.64%, which was higher than that of free Cur (*p* < 0.05). Moreover, NCs prepared by using PC, vanillin, and Lys also showed good antioxidant capacity from 1 to 40 μg/mL. As for the IC_50_ values, Cur NPs showed a more efficient radical-scavenging capacity than that of Vc, Cur, and NCs (Table S1).

Green ABTS^+^ radicals, formed by the reaction between ABTS and potassium persulfate, have strong absorption in the visible region at 734 nm [30]. As shown in Figure 4b, the scavenging ability of Vc, Cur, NCs, and Cur NPs increased with increasing concentration. At a concentration of 25 μg/mL of Cur or NCs, the free radical scavenging percentage of NCs alone reached 76.54%, while that of Cur NPs was 83.38%, both of which were significantly higher than that of free Cur and the positive control—Vc. Moreover, the IC_50_ value was lower than that of Vc, Cur, and NCs. Therefore, it could be seen that NCs alone showed good antioxidant ability, and Cur NPs obtained by loading Cur with these nanocarriers also had strong antioxidant ability.

### 3.5. Photothermal Stability Analysis

Cur, as a bioactive compound with strong photothermal sensitivity, has diketone groups and phenolic hydroxyl groups in its structure that are easily decomposed under the interference of external factors, thus affecting its biological activity. Therefore, the effects of UV radiation and temperature on the stability of loaded Cur were investigated to evaluate the potential advantages of encapsulation.

**Figure 5:**
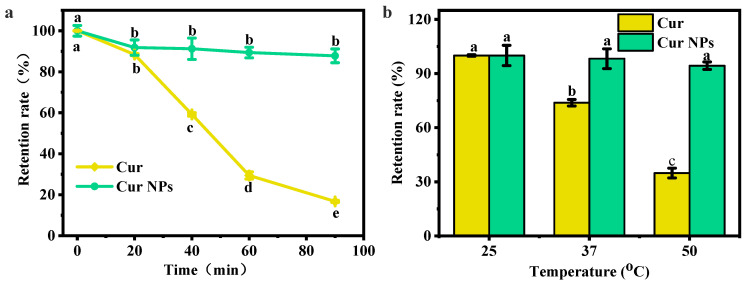
(**a**) UV irradiation and (**b**) thermal stability analyses for Cur and Cur NPs. Note: the lower letters a, b, and indicate that there are statistically significant differences between the samples.

As shown in Figure 5a, the content of free Cur and embedded Cur presented a downward trend with the prolongation of irradiation time, and the degradation rate of free Cur was relatively faster. When the radiation time was 90 min, the content of free Cur was only 16.75%, while the content of Cur in the nanoparticles was still as high as 87.77%, indicating that the photostability of Cur was significantly improved after embedding. This was because most Cur molecules was embedded inside PC nanocarriers, and the carriers acted as an outer shell to isolate Cur from direct contact with UV light and high temperature [31]. The results of the thermal stability experiment also showed a similar trend (Figure 5b). For example, the retention rate of Cur was 35% at 50 °C, and the retention rate of embedded Cur was 94%, demonstrating an increase of 59%. These results indicated the load of Cur in nanoparticles effectively enhanced their photothermal stability.

### 3.6. In Vitro Release Profile Analysis

**Figure 6:**
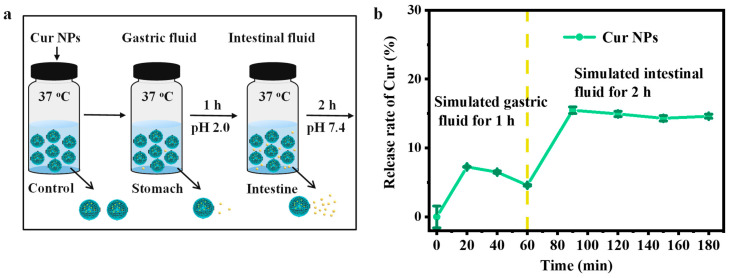
(**a**) Schematic diagram of simulated digestion and (**b**) release profile of Cur NPs.

The release profile of Cur-loaded nanoparticles was evaluated using artificial gastric and intestinal fluids (Figure 6a). In the simulated gastric fluid, the release of Cur from the nanoparticles was less than 10%, and the release curve presents a rising and then decreasing trend (Figure 6b). This was because at a pH of 2.0, the released Cur had low water solubility, and Cur could precipitate as the incubation time was prolonged, resulting in a decrease in its absorbance. When transferred to the simulated intestinal fluid, it was found that the release rate of Cur was significantly improved, which allowed Cur to quickly reach the effective concentration and achieve slow release in the intestinal tract.

### 3.7. Cytotoxicity Analysis

PC12 cells, a cell line originated from a pheochromocytoma of rat adrenal medulla, have typical neuronal and high-level glucocorticoid receptor-producing properties, which make them widely applied as a common cell model for studying neuronal functions and neurological diseases [32,33]. CORT is a type of glucocorticoid produced by the adrenal cortex, and its excessive presence in the body is associated with the occurrence and development of neurological diseases, for example, depression [34]. Continuous exposure to high levels of CORT can cause a series of pathological changes in PC12 cells, such as decreased cell viability and excessive production of reactive oxygen species [35]. Therefore, a CORT-induced PC12 cell model can be used to analysis the intervention function of bioactive compounds and their potential therapeutic potential in preventing or treating depression.

**Figure 7:**
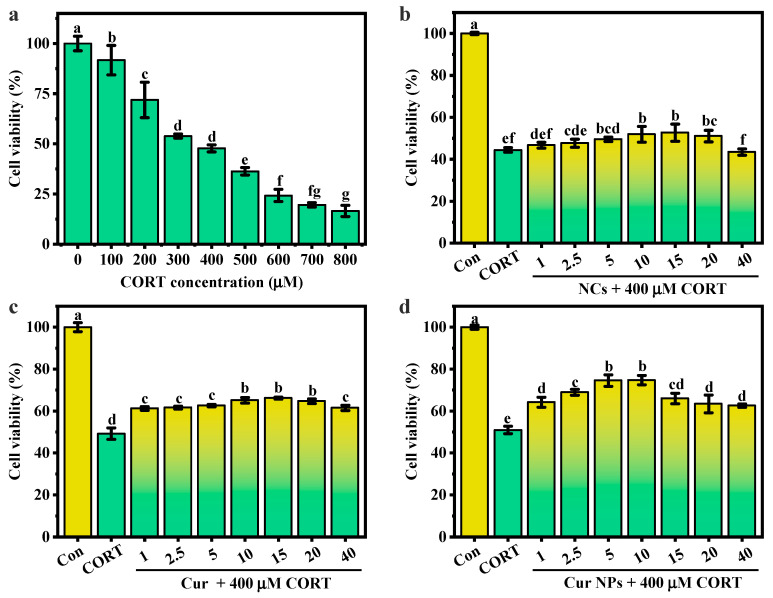
Cell viability analysis. (**a**) The effect of different concentrations of CORT on the viability of PC12 cells. Effect of (**b**) NCs, (**c**) Cur, and (**d**) Cur NPs on the cell viability of 400 μM CORT-induced PC12 cells. Note: the lower letters a−e indicate that there are statistically significant differences between the samples.

PC12 cells were induced by different contents of CORT, and the degree of cell damage caused by CORT was assessed by detecting cell viability. As shown in Figure 7a, with an increase in CORT concentration, the viability of PC12 cells significantly decreased in a dose-dependent way. When the concentration of CORT was 400 μM, the viability decreased to 47.73%, and this concentration was subsequently selected to induce cell damage. Different concentrations of Cur, NCs, and Cur NPs were co-cultured with PC12 cells for 24 h, and then 400 μM CORT was used to induce cell damage. The results in Figure 7b showed that with an increase in NC content, cell viability presented a first increasing and then decreasing trend. At a concentration of 15 μg/mL, the cell viability of cells treated with NCs reached its maximum level of 52.67%, which was 8.23% higher than that of the CORT-induced cell model. NCs and Cur NPs also presented a similar trend after the intervention, with the highest cell viability of 66.15% at 15 μg/mL for Cur and 74.74% at 10 μg/mL for Cur NPs (Figure 7c,d). This indicated that Cur NPs had a better intervention effect on PC12 cell injury induced by CORT at lower concentrations.

### 3.8. Intracellular Antioxidant Capacity Analysis

Oxidative stress has an important effect in CORT-induced PC12 cell damage [36]. The intervention effect of the prepared Cur NPs on PC12 cells was assessed by measuring the levels of T-SOD, CAT, and MDA.

**Figure 8:**
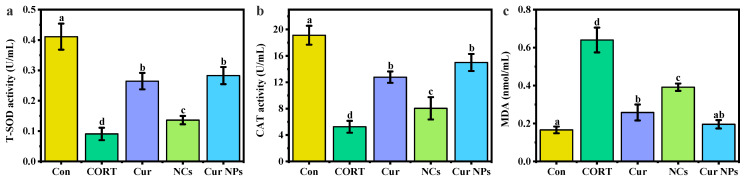
Effect of Cur, NCs, and Cur NPs on the levels of (**a**) T-SOD, (**b**) CAT, and (**c**) MDA in PC12 cells induced by CORT. Note: the lower letters a, b, c, and d indicate that there are statistically significant differences between the samples.

As shown in Figure 8a, the level of T-SOD, an important antioxidant enzyme, was significantly reduced after induction by 400 μM CORT, and the T-SOD level increased by 42.30%, 11.06%, and 46.77% after intervention with Cur, NCs, and Cur NPs. The level of CAT also showed a similar trend, and Cur NPs had a relatively better intervention effect on CORT-induced PC cells (Figure 8b). The content of MDA, the end product of lipid oxidation, can also be used as one of the indexes to investigate the severity of cells damaged by CORT. Compared with the CORT-treated group, the production of MDA was significantly reduced after pretreatment with Cur, NCs, and Cur NPs (Figure 8c). This showed that NCs had a protective effect on cell damage caused by CORT, and Cur NPs, after loading with Cur, showed a stronger intervention effect, indicating that the combination of Cur and NCs could exert a synergistic effect.

### 3.9. Blood Compatibility Analysis

**Figure 9:**
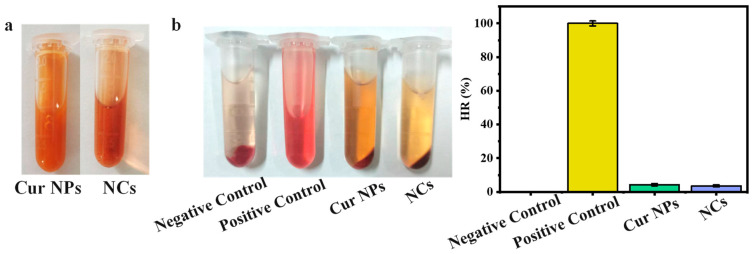
(**a**) Optical images of Cur NPs and NCs. (**b**) Optical images and hemolysis rate (HR) of negative control, positive control, Cur NPs, and NCs after treatment of red blood cells.

In this study, hemolysis experiments were employed to evaluate the biocompatibility of the prepared nanoparticles. As shown in Figure 9a, the prepared Cur NPs and NCs solutions showed yellowish-brown and reddish-brown colors, respectively, due to the presence of Cur and PCs. Subsequently, the samples were incubated with red blood cells. The blood samples co-incubated with TritonX−100 showed a clear red solution compared with the saline negative control, indicating that red blood cells were ruptured and hemolysis occurred (Figure 9b). After co-incubation with Cur NPs and NCs, the color was similar to that of the sample itself, and the hemolysis rate was less than 5%, indicating that the prepared Cur NPs and NCs had good biocompatibility.

## 4. Conclusions

Cur, as a plant polyphenol, has antioxidant, anti-inflammatory, neuroprotective, and other health benefits, but its development is limited due to shortcomings such as poor water solubility, stability, and bioavailability. Therefore, the purpose of this study was to design and prepare functional nanocarriers to load Cur, improve stability and availability, and even maximize the biological effects of Cur. Firstly, PCs, amino acids, and vanillin were used as raw materials to prepare nanocarriers through the Mannich reaction. By adjusting the concentration of PCs (100, 200, and 300 mg/50 mL) and vanillin (10, 13, 20, and 40 mg/50 mL), and the types of amino acids (Lys, Glu, or Cys), nanocarriers with controlled size and antioxidant properties were obtained. When the concentrations of PCs, vanillin, and Lys were 200, 20, and 50 mg/50 mL, respectively, the obtained NCs−1 had the smallest particle size of 328.47 nm. The molecular interaction among the phenolic hydroxyl groups of PCs, aldehyde groups of vanillin, and amino groups of amino acids was proven by FTIR, UV−vis, and XRD spectra. Subsequently, this nanocarrier was used to load Cur. The embedding efficiency of Cur NPs obtained by an anti-solvent precipitation method was 85.97%, and the morphology was uniform and square. Through FTIR and UV−vis spectroscopy analysis, Cur was found to form hydrophobic nuclei after being loaded into the nanocarriers, resulting in its characteristic peaks being reduced or masked. The XRD spectra of Cur NPs had the same crystal diffraction peaks of Cur, and these results all demonstrated that Cur was successfully loaded into the nanocarriers. The antioxidant experimental results showed that compared with Vc, the prepared NCs had stronger DPPH and ABTS radical scavenging abilities. Cur NPs obtained by loading Cur had relatively stronger antioxidant properties and could achieve a 50% radical-scavenging effect at low concentrations. Subsequently, the photothermal stability of Cur and Cur NPs under different conditions was compared, and it was found that the content of the loaded Cur was still as high as 87% under the protection of NCs. The simulated digestion results showed that Cur NPs were slowly released in the intestinal tract. Finally, a CORT-induced PC12 cell model was used to evaluate the biocompatibility and activity of Cur NPs. Compared with CORT-induced cells, the cell viability after intervention with Cur NPs (10 μg/mL) increased by 23.77%. The results of hemolysis experiments also proved that the prepared nanoparticles had good blood biocompatibility and could effectively improve the oxidative stress damage of cells caused by CORT. This study provides a theoretical basis for the application of bioactive compounds in neurodegenerative diseases.

## Figures and Tables

**Table 1:** Different concentrations of PCs, vanillin, and amino acids for the fabrication of nanocarriers named NCs−1~NCs−8.

Sample Name	PCs (mg/50 mL)	Vanillin (mg/50 mL)	Types of Amino Acid	Particle Size (nm)	PDI
NCs−1	200	20	Lys	328.47 ± 6.00	0.37 ± 0.25
NCs−2	200	20	Glu	877.87 ± 72.95	0.29 ± 0.02
NCs−3	200	20	Cys	952.99 ± 18.10	0.40 ± 0.04
NCs−4	100	20	Lys	463.96 ± 207.39	0.43 ± 0.04
NCs−5	300	20	Lys	337.44 ± 24.50	0.41 ± 0.03
NCs−6	200	10	Lys	734.63 ± 161.82	0.44 ± 0.07
NCs−7	200	13	Lys	601.48 ± 49.91	0.46 ± 0.01
NCs−8	200	40	Lys	397.02 ± 19.66	0.37 ± 0.02

Note: Lys, lysine; Glu, glutamic acid; Cys, cysteine.

## Data Availability

The original contributions presented in this study are included in the article/Supplementary Material; further inquiries can be directed to the corresponding author.

## Supplementary Materials

The following supporting information can be downloaded at https://www.mdpi.com/article/10.3390/foods13233958/s1, Table S1: The half inhibitory concentration (IC_50_) values of DPPH and ABTS scavenging abilities of Vc, Cur, NCs, and Cur NPs.
