# Supplementary material for: Preparation and Characterization of Small-Size and Strong Antioxidant Nanocarriers to Enhance the Stability and Bioactivity of Curcumin
Source: Foods. 2024 Dec 8;13(23):3958. doi: 10.3390/foods13233958 (PMC11641333; doi:10.3390/foods13233958)
Supplement: Supplementary file 1 [file foods-13-03958-s001.zip › foods-3313879-supplementary.pdf]

Preparation and Characterization of Small-Size and Strong Antioxidant Nanocarriers to Enhance the Stability and Bioactivity of Curcumin

Shanshan Tie[1], Yujin Yang[1], Jiawei Ding, Yanyan Li, Mengmeng Xue, Jianrui Sun, Fang Li, Qiuxia Fan, Ying Wu, Shaobin Gu*

College of Food and Bioengineering, Henan University of Science and Technology, Luoyang 471023, China

*Corresponding authors: Shaobin Gu, shaobingu@haust.edu.cn

Table S1. The half inhibitory concentration (IC_{50}) values of DPPH and ABTS scavenging ability of Vc, Cur, NCs, and Cur NPs.

IC_{50} (μg/mL)	Vc	Cur	NCs	Cur NPs
DPPH radical	40.9940	6.5480	5.5660	4.5580
ABTS radical	36.7620	34.7840	14.2610	5.5470
